# Endoscopic retrieval of multiple sewing needles by using the tip of a magnetic tube

**DOI:** 10.1016/j.vgie.2021.01.007

**Published:** 2021-02-24

**Authors:** Shinichi Morita, Kazuyoshi Yagi, Takahiro Hoshi, Satoshi Abe, Shuji Terai

**Affiliations:** 1 Department of Gastroenterology and Hepatology, Uonuma Institute of Community Medicine Niigata University Hospital, Niigata, Japan; 2 Department of Gastroenterology and Hepatology, Niigata University Hospital, Niigata, Japan

## Abstract

Video 1 Multiple sewing needles are retrieved from the stomach endoscopically using the tip of a magnet tube.

Multiple sewing needles are retrieved from the stomach endoscopically using the tip of a magnet tube.

Ingested needles can easily penetrate the GI wall and cause serious conditions such as peritonitis. They can also migrate to other organs.^1^^,^^2^ Thus, prompt endoscopic retrieval is necessary. Generally, forceps are used to retrieve ingested needles,^3^^,^^4^ but if the needle is not properly grasped, its tip may damage the digestive tract. Moreover, food residues make needle retrieval more challenging. We report a case in which sewing needles were easily retrieved from the stomach endoscopically using the tip of a magnetic tube, which is mainly used in children when retrieving swallowed metallic objects from the stomach under fluoroscopy^5^ (Fig. 1).

**Figure 1:**
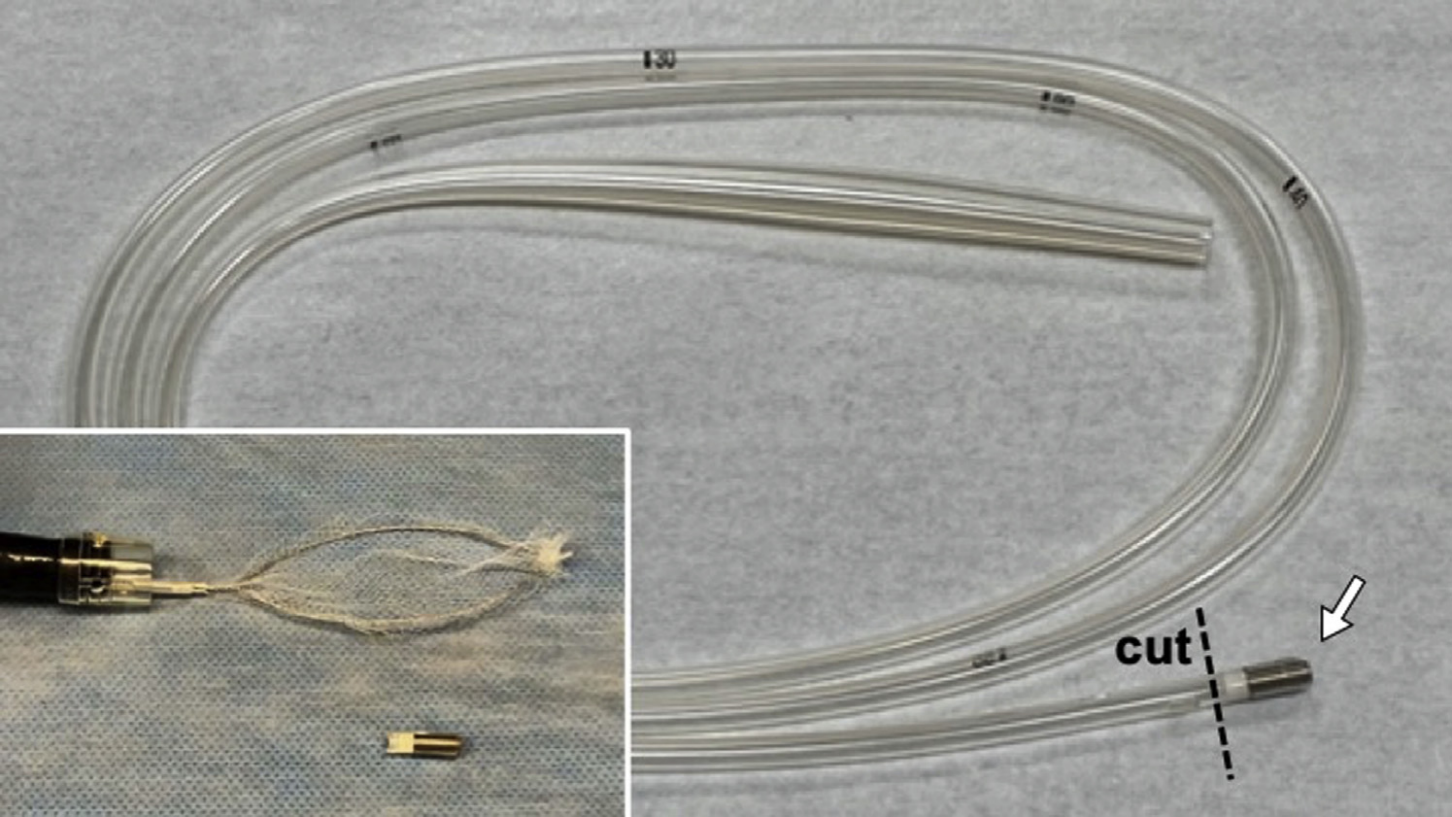
Magnet tube (Argyle; Covidien, Tokyo, Japan). A magnet (*arrow*) is inserted into the tip of the polyvinyl chloride tube. Insets show the tip of the magnet tube and a net catheter that is preinserted through the endoscope.

A 23-year-old woman with schizophrenia was referred to our hospital with abdominal pain after she swallowed multiple sewing needles, intending to self-harm. Abdominal CT showed needles in the stomach and duodenum, some of which had penetrated the GI wall (Fig. 2A and B).

**Figure 2:**
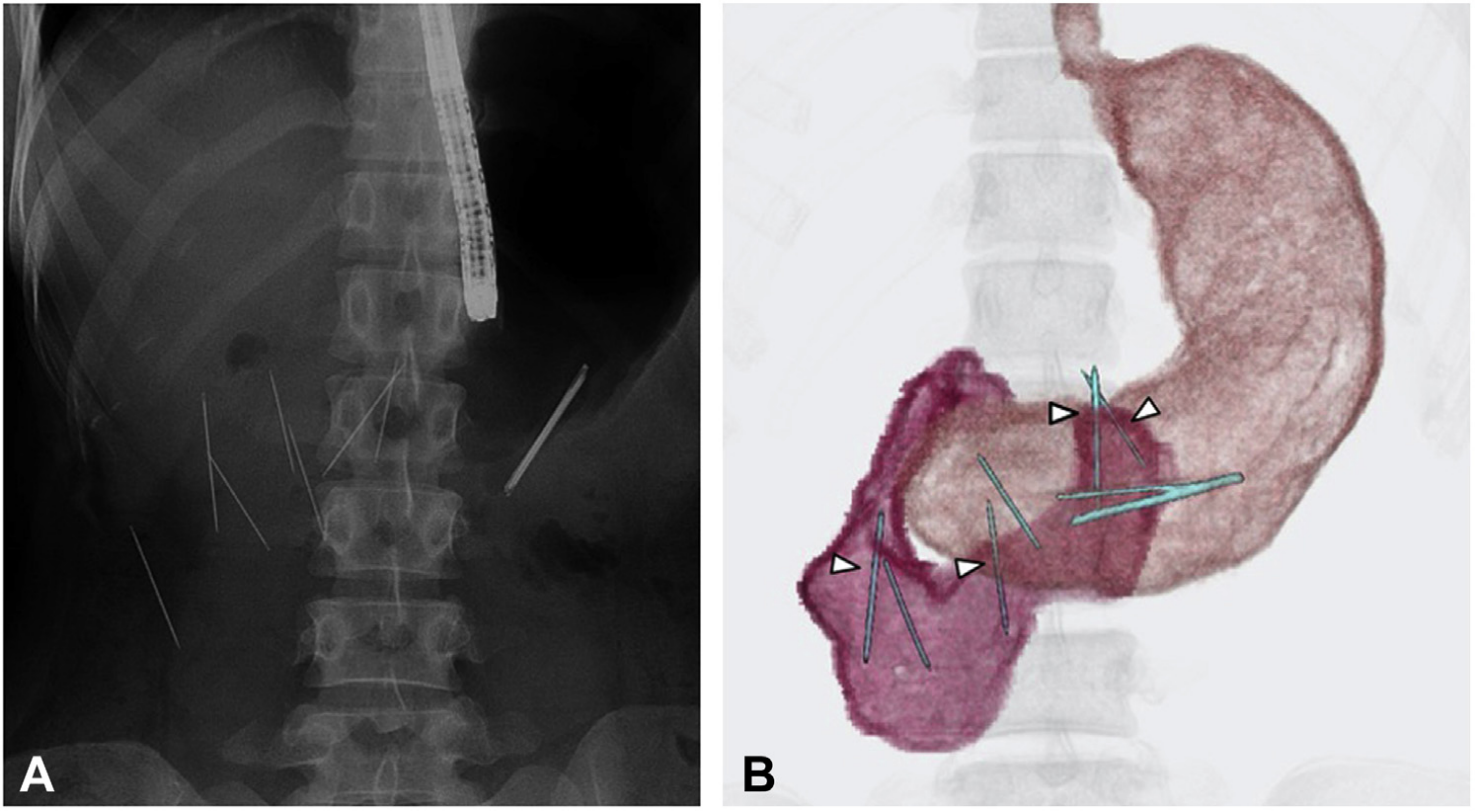
**A,** Fluoroscopic images showing multiple sewing needles in the stomach and duodenum. **B,** Upper GI images by CT reconstruction show multiple needles in the stomach and duodenum. Some are penetrating the duodenal wall (*arrowheads*).

An EGD was performed to retrieve the needles (Video 1, available online at www.giejournal.org). First, the needles penetrating the duodenal wall were removed with forceps and carried into the stomach. The tip of a magnetic tube (Argyle; Covidien, Tokyo, Japan) was cut and captured using a net catheter (Olympus Medical, Tokyo, Japan) and preinserted through an endoscope (GIF-Q260J; Olympus). While the patient was sedated, an overtube was inserted to prevent aspiration. The endoscope was reinserted into the stomach, and the magnet was placed near the needles using fluoroscopy. Multiple needles were attached to the magnet at one time and easily retrieved from the body (Figs. 3 and 4). This operation was repeated several times, and all 16 needles were retrieved with no adverse events (Fig. 5). The procedure took 20 minutes.

**Figure 3:**
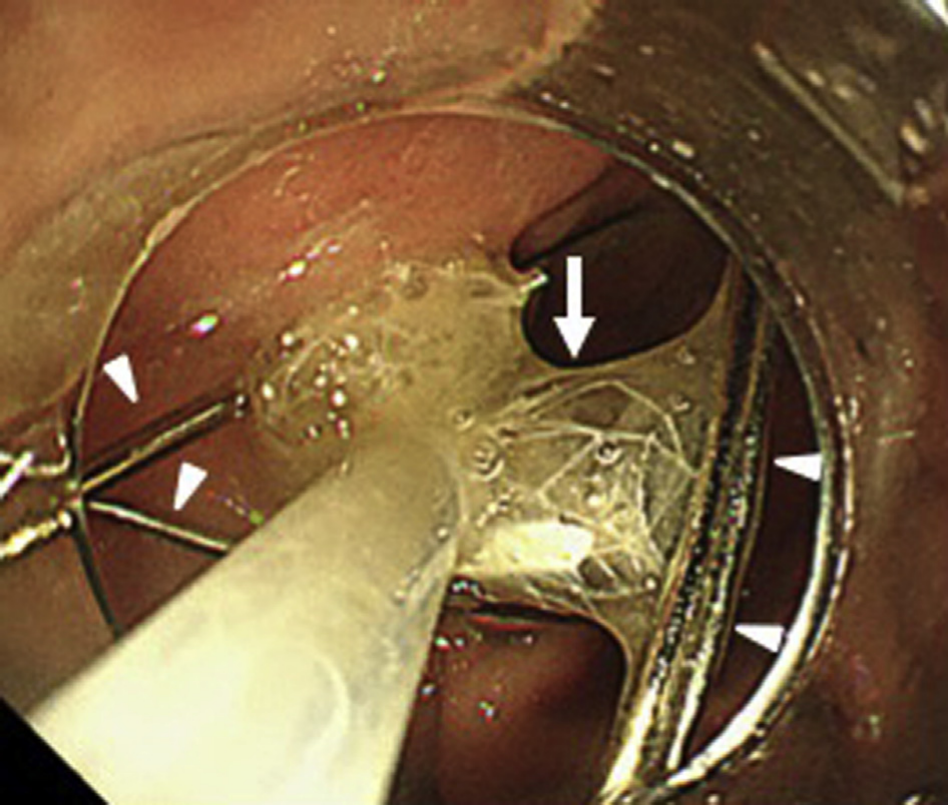
Fluoroscopy shows several sewing needles (*arrowheads*) attached to the magnet (*arrow*).

**Figure 4:**
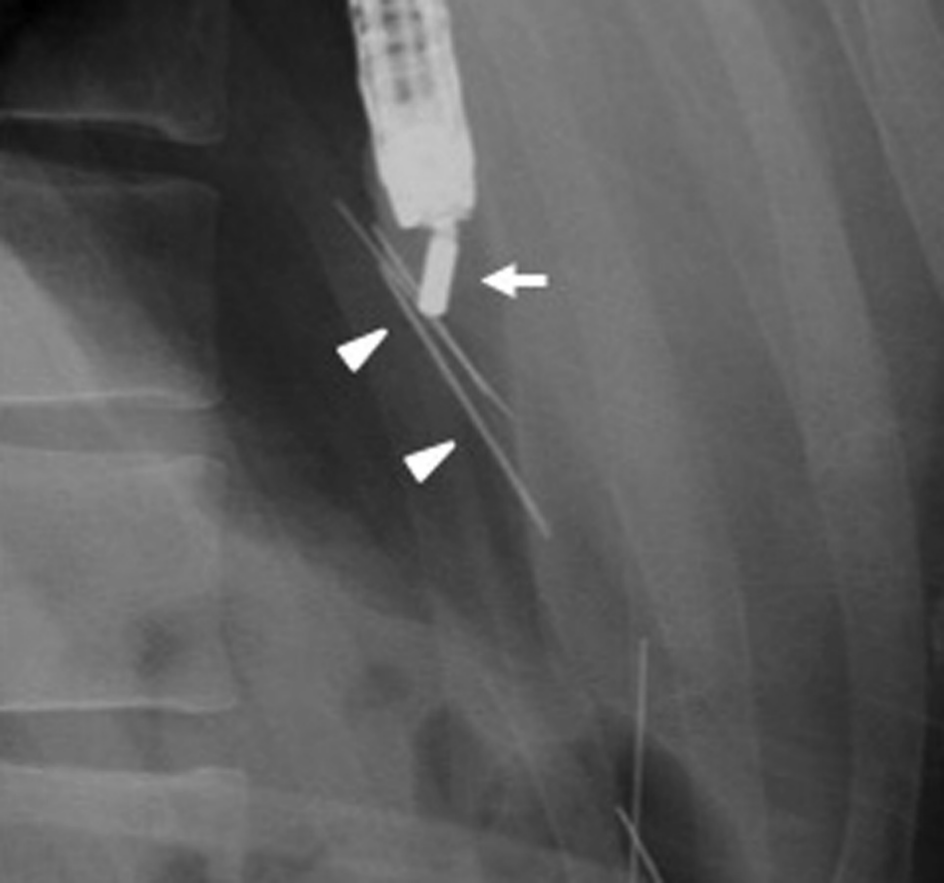
The magnet is captured using the retrieval net catheter (*arrow*). Several needles are attached to the magnet (*arrowheads*).

**Figure 5:**
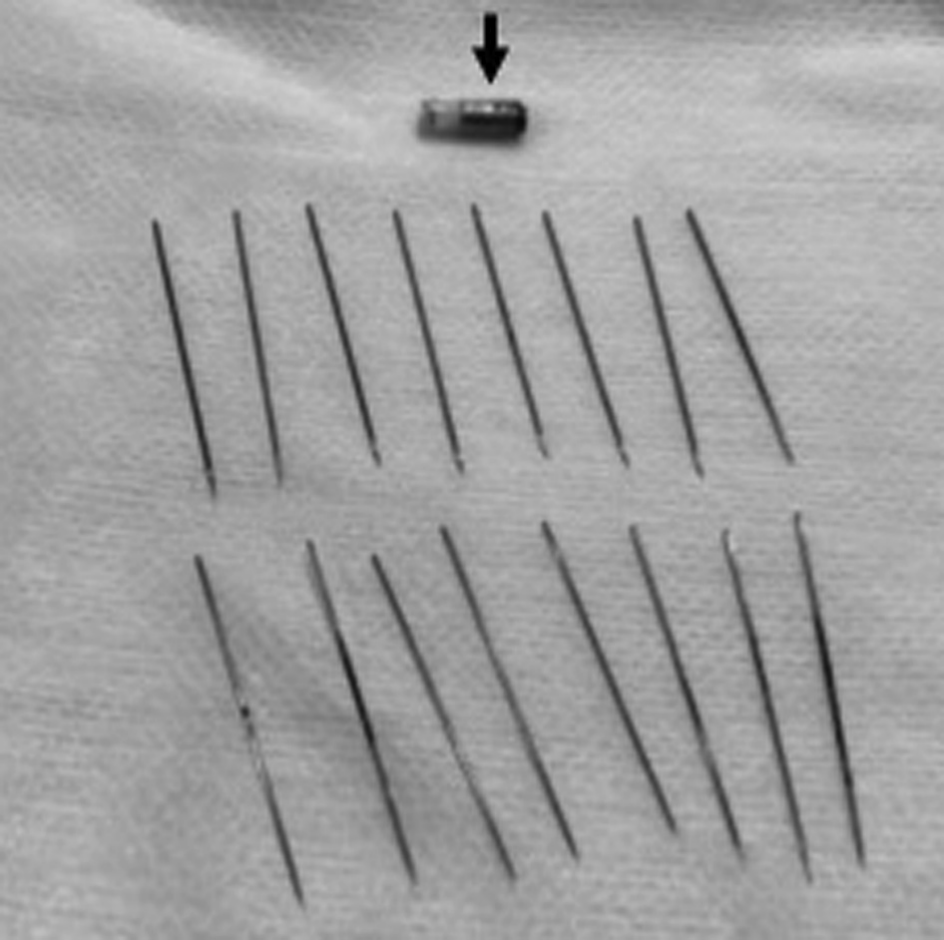
Retrieval of 16 sewing needles and the tip of the magnet (*arrow*).

Multiple needles can be retrieved easily with this technique, even if they have food residue. Moreover, the needles can be detached even when they hit the GI wall. Importantly, the magnet attachment can be easily shifted to ensure that the needle is safely retrieved. Therefore, swallowed needles can be easily retrieved while minimizing damage to the GI wall.

## Disclosure

*All authors disclosed no financial relationships.*

## Supplementary data

Video 1 Multiple sewing needles are retrieved from the stomach endoscopically using the tip of a magnet tube.
